# Factors and Disparities Influencing Sodium-Glucose Cotransporter 2 Inhibitors and Glucagon-like Peptide 1 Receptor Agonists Initiation in the United States: A Scoping Review of Evidence

**DOI:** 10.3390/pharmacy13020046

**Published:** 2025-03-19

**Authors:** Josiah Moore, Ndidi Iheme, Nicholas S. Rebold, Harriet Kusi, Constance Mere, Uzoamaka Nwaogwugwu, Earl Ettienne, Weerachai Chaijamorn, Dhakrit Rungkitwattanakul

**Affiliations:** 1 Department of Clinical and Administrative Pharmacy Science, Howard University College of Pharmacy, Washington, DC 20059, USA; 2 Department of Pharmacy, MedStar Georgetown University Hospital, Washington, DC 20007, USA; 3 Division of Nephrology, Department of Medicine, Howard University College of Medicine, Washington, DC 20059, USA; 4 Department of Pharmacy Practice, Faculty of Pharmaceutical Sciences, Chulalongkorn University, Bangkok 10330, Thailand

**Keywords:** disparity, medication, minority, initiation

## Abstract

Introduction: Health disparities affecting minority populations and resulting in poorer outcomes for disadvantaged groups have been documented in the literature. Sodium/glucose-cotransporter 2 (SGLT2i) inhibitors and GLP-1 receptor agonists (GLP-1RA) markedly decrease mortality from kidney and cardiovascular events. However, little is known about the factors and disparities that lead to differences in SGLT2i and GLP-1RA initiation across different ethnic groups. Methods: This scoping review queried databases using key terms related to disparities in the initiation of SGLT2i and GLP-1RA among high-risk populations. Relevant data from eligible studies were extracted, organized, and analyzed thematically to identify key trends and patterns in the literature. Result: Nineteen studies were included in this review. Key risk factors influencing uptake included age, provider type, race, sex, education, comorbidities, insurance, and income, with minority patients consistently showing lower rates of initiation due to systemic barriers and socioeconomic disparities. Patients who were younger, male, had higher education or income levels, and received care from specialists were more likely to use these therapies. Conclusion: The adoption of SGLT2i and GLP-1RA remains suboptimal despite their proven kidney and cardiovascular benefits. Targeted efforts to reduce socioeconomic and racial inequities based on the factors identified should be encouraged.

## 1. Introduction

Health disparities are known to have a vast impact on marginalized groups within the United States (US) healthcare system, resulting in poorer outcomes [1]. This is a phenomenon that is in large due to the lack of access to adequate care, lack of provider education, and structural racism that exist within the healthcare delivery system [2]. The medication treatment to limit kidney disease progression and reduce cardiovascular mortality has significantly progressed in the past decade. Additionally, a 2008 US Food and Drug Administration (US FDA) mandate requiring clinical trials to assess the cardiovascular safety of all new drugs has led to a wealth of high-quality data regarding their risks and benefits [3]. Clinical data demonstrated that newer diabetes medications—glucagon-like peptide 1 receptor agonists (GLP-1RAs) and sodium/glucose cotransporter 2 inhibitors (SGLT2is)—offer kidney and cardiovascular protective benefits [4]. However, despite strong evidence supporting their advantages, there is limited research on prescribing patterns for these medications, particularly among populations at high risk for health disparities. Nevertheless, it is clear that uptake has been significantly lower amongst minority populations. Therefore, we aim to examine the evidence regarding the uptake of SGLT2is and GLP-1RAs. We also aimed to identify risk factors influencing the uptake of these medications and how these factors are defined in the literature for several ethnic groups in the United States. We hope to provide solutions to enhance adoption amongst the general population based on our findings.

## 2. Methods

A scoping review was carried out by querying the PubMed, Google Scholar, and MEDLINE electronic databases, following the Preferred Reporting Items for Systematic Reviews and Meta-Analysis Extension for Scoping Reviews (PRISMA-ScR) guidelines [5]. We also enhanced the search by using an artificial intelligence (AI)-powered search engine (Consensus) to ensure the robustness of our results.

### 2.1. Seach Strategy

A search was conducted on the aforementioned databases from inception to October 2024. The search for articles was conducted in the English language. The key terms used were as follows: (“Race” OR “ethnicity” OR “socioeconomic status”) AND (“Black” OR “Hispanic” OR “Hispanics” OR “minority” OR “minoritized population” OR “African Americans” OR “African American”) AND (“diabetes mellitus” OR “DM” OR “chronic kidney disease” OR “CKD” OR “heart failure” OR “HF”) AND (“SGLT2i” OR “GLP-1RAs” OR “empagliflozin” OR “dapagliflozin” OR “canagliflozin” OR “liraglutide” OR “semaglutide” OR “dulaglutide”) AND (“disparities” OR “uptake” OR “prescribing pattern” OR “use” OR “prescribe” OR “adopt” OR “adoption” OR “dispense” OR “dispensing”).

### 2.2. Article Selection

**Figure 1:**
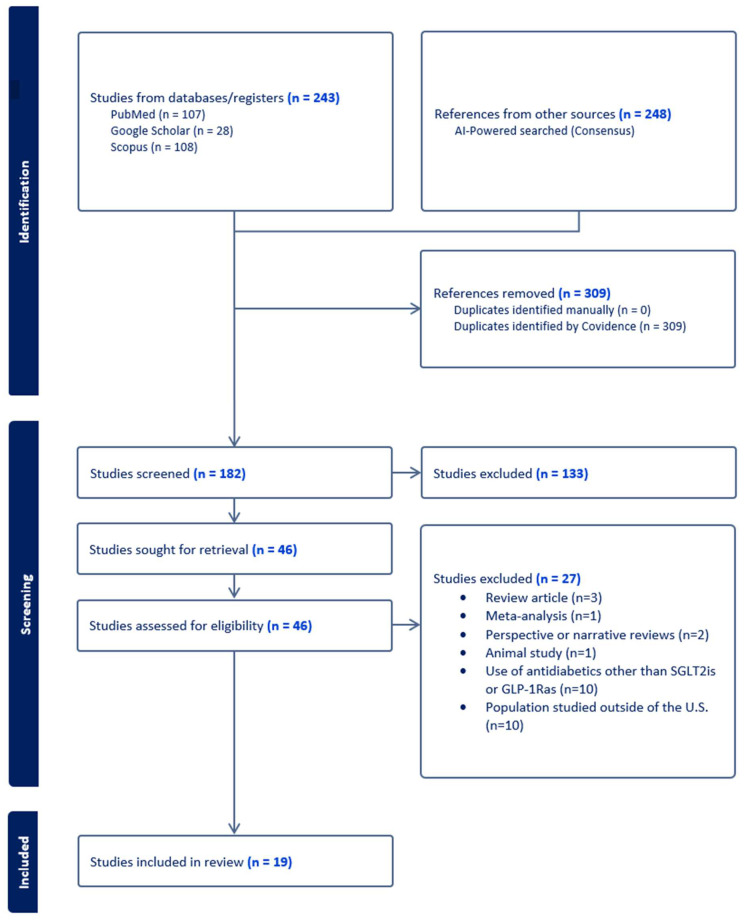
Preferred Reporting Items for Systematic Reviews and Meta-Analyses (PRISMA) flowchart.

Article evaluation began with a first screening in which two researchers independently assessed all articles by title and abstract using Covidence systematic review software (Veritas Health Innovation Melbourne, Victoria, Australia), and articles were included (Figure 1) if they satisfied the inclusion and exclusion criteria shown in Table 1.

The two researchers then independently reviewed and analyzed the full-text articles to determine their eligibility for inclusion. Any disagreements regarding article selection were resolved through discussion and consensus within the team. Finally, relevant data were extracted and organized into tables for comparative analysis, while the final set of articles was examined thematically to identify key trends and patterns in the literature. To ensure the validity and generalizability of research findings, the kappa statistic was used to evaluate the agreement between two reviewers for full-text review. A kappa score of 0.6 or greater suggests substantial agreement between reviewers [6].

Our scoping review was conducted to answer the following questions:

1. What is the current evidence available on the disparities that impact SGLT2i/GLP-1RA uptake across study participants?
2. What are the risk factors that impact the uptake of SGLT2is/GLP-1RAs and how are they defined in the literature amongst the general population in the United States?
3. Has there been published research providing solutions to improve the initiation and uptake of SGLT2is/GLP-1RAs amongst the general population in the United States?
4. What are the gaps identified?

### 2.3. Data Extraction and Analysis

Data extraction pertaining to the research questions was performed by JM and verified by DR via an independent data extraction software using Microsoft Word Version 2502 (Microsoft Corporation, Redmond, WA, USA). Key information related to the article, including the year of publication, journal title, first author’s information, sample size, and study design, was extracted and input into an independent data extraction depository. Information related to SGLT2i and/or GLP-1RA uptake (findings, factors, solutions) was recorded. Key findings from each included article were extracted by JM and reviewed by all authors according to the aforementioned research questions. Due to the heterogeneity of the studies, no statistical analysis was performed; rather, a narrative approach was utilized to summarize the literature.

### 2.4. Methodological Quality Assessment

The included studies were individually assessed via full-text review for methodological quality by the two reviewers independently (JM and DR). The quality assessment was performed by the STROBE checklist. Studies were stratified into high-, moderate-, and low-quality based on the scores [7]. The scores were not used to include or exclude the studies for review but were instead used to describe the quality of evidence.

## 3. Results

The initial search yielded 243 studies from PubMed, Google Scholar, and Scopus. Consensus yielded 248 studies. Of those, 309 duplicates were removed in Covidence. Following this, a first screening of the article sample was conducted, and 182 articles were assessed (Figure 1). During the first screening phase, 133 articles were excluded, as they did not meet the inclusion criteria. Next, a full-text review of all 46 remaining articles was performed. Of those, 27 were excluded, resulting in only 19 relevant articles that satisfied the inclusion criteria. There was an acceptable level of screening agreement among investigators (kappa = 0.51).

Research Question 1:

1. What is the current evidence available on the disparities that impact SGLT2i/GLP-1RA uptake across study participants?

Table 2 summarizes the characteristics of the articles included. In total, 19 studies were included in the characteristics of the analysis. Approximately two-thirds (68.3%) of the articles were published between 2021 and 2023. The majority (*n* = 12; 63.2%) utilized a retrospective cohort design. The 19 citations, representing a total of approximately 12,403,354 patients, were published across 13 different journals. The majority of the studies were published in the *Journal of Managed Care & Specialty Pharmacy* (21%) and *The Journal of the American Medical Association* (21%). The first SGLT2i approved was canagliflozin, in March of 2013. Furthermore, the first GLP-1RA, Byetta (exenatide), was approved in 2005. However, when it comes to examining disparities associated with the impact of the uptake of these medicines, the first paper included in our analysis was published in 2019, which shows a lag in disparity studies. For this analysis, all studies were published in the United States.

2. What are the risk factors that impact the uptake of SGLT2is/GLP-1RAs and how are they defined in the literature amongst the general population in the United States?

**Figure 2:**
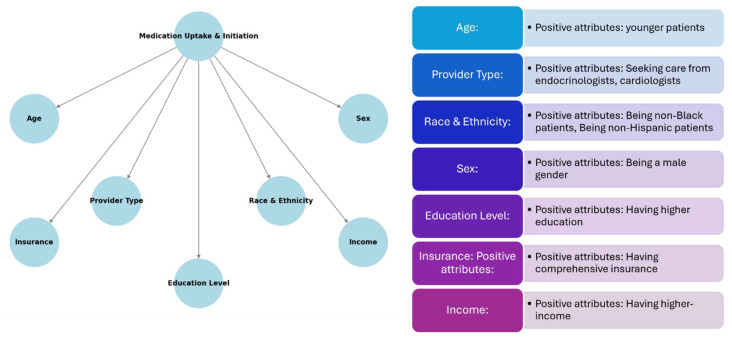
Overall summary of results and study findings.

Several risk factors that impact the uptake of SGLT2is and GLP-1RAs were identified. These risk factors include age, provider type, race, sex, education, comorbidity status, insurance type, and income. Patients using SGLT2is/GLP-1RAs were significantly younger across multiple studies analyzed. Researchers concluded that younger patients may be more adherent to these medications and able to tolerate them better than older individuals. Provider type was examined across multiple studies, and many concluded that patients that receive care from a specialist (endocrinologist) are more likely to receive a prescription for SGLT2is/GLP-1RAs compared to patients that receive care from a primary care physician. Differences in race were also examined in 16 of 19 studies included in the analysis. Black patients had significantly lower rates of SGLT2i/GLP-1RA uptake compared to their White counterparts. Sixteen articles stated that racial bias largely contributes to this phenomenon. When sex was analyzed, in the majority of studies, men were more likely to initiate these medications than women. Many studies cited side effects as a concerning factor, especially with SGLT2is, which are known to cause urinary tract infections (UTIs). Education was examined in only 4 of 19 studies included in the analysis. These articles cited that patients living in neighborhoods with higher education levels were more likely to receive newer second-line diabetes medications such as SGLT2is/GLP-1RAs. These articles also mentioned that the provider bias that exists around education is present due to the belief that patients that are educated are more adherent, which is essential with high-cost medications. Many of the comorbidities that were noted across study participants were high atherosclerotic cardiovascular disease (ASCVD), CKD, and heart failure with reduced ejection fraction (HFrEF). The majority of studies that included comorbidities in their results concluded lower rates of prescriptions of SGLT2is and GLP-1RAs amongst these patients, despite the overwhelming evidence of cardiovascular and kidney protection from these medications. Patients that were commercially insured were significantly more likely to receive SGLT2is/GLP-1RAs vs. uninsured/underinsured patients. When it comes to income, patients with lower ZIP code-linked household income were independently associated with lower rates of initiation of novel therapies such as SGLT2is and GLP-1RAs. The key findings of each study are listed in Table 3 and Figure 2.

3. Has there been published research providing solutions to improve initiation and uptake of SGLT2i in the general population?

The articles included in our analysis focused on risk factors that contribute to the underutilization and lack of uptake of SGLT2is and GLP-1RAs. None of the articles analyzed stated a potential solution to the lack of adoption of these medications.

4. What are the gaps identified?

Studies included in this scoping review analysis uncovered racial and ethnic disparities in the uptake of SGLT2is and GLP-1RAs. Nevertheless, there are gaps within the literature. First, all studies were not experimental; rather, all of them were observational. This may limit the generalizability of study conclusions. Second, we intended to only include studies that were conducted in the US since this would best represent current contemporary issues. However, this might impact the generalizability of the findings to other countries or regions where the population is homogeneous. Conversely, our findings might be beneficial to countries or regions that share similar patient demographics to those in the US. Third, many studies reported factors affecting SGLT2i and GLP-1RA initiation. However, the populations that were studied were largely different, creating difficulty in drawing conclusions and calling for larger-scale or multicenter studies to limit geographical differences. Fourth, of all studies that established factors affecting SGLT2i/GLP-1RA initiation, there were no studies recommending a potential solution to improve the uptake among patient population at risk. Research should further focus on interventions to improve the initiation and effectiveness of the intervention, rather than focusing on just establishing risk factors.

## 4. Study Quality

Methodological study quality was assessed using the STROBE criteria [7]. Of the nineteen studies analyzed, ten studies were graded as ‘low-quality’, nine studies as ‘moderate-quality’, and no studies were graded as ‘high-quality’ (Table 4).

## 5. Discussion

It is no secret that there is a huge need for the uptake of SGLT2is and GLP-1RAs specifically for the purpose of kidney and/or cardiovascular protection [27]. Despite these compelling benefits, real-world uptake remains suboptimal, leaving many patients without access to therapies that could significantly reduce morbidity and mortality [28]. This gap is particularly concerning because kidney and cardiovascular complications are leading causes of hospitalization and death, further burdening healthcare systems [29]. Increasing the use of SGLT2is and GLP-1RAs represents a critical opportunity to address these unmet needs, improve patient outcomes, and reduce healthcare costs by preventing complications such as end-stage kidney disease, heart failure exacerbations, and cardiovascular events like heart attacks and strokes [30]. Thus, prioritizing the broader adoption of these therapies is essential for both patient and healthcare system benefit [30]. This paper focuses on the critical role of secondary and tertiary prevention in addressing noncommunicable diseases, highlighting the importance of these therapies in mitigating disease progression and preventing complications.

Age plays a crucial role in the uptake and initiation of SGLT2is and GLP-1RAs for several reasons [26]. While older adults often have more regular healthcare visits which can facilitate education about medication and the benefits of adherence, younger individuals may be perceived as better candidates due to their ability to tolerate side effects, their potential for long-term kidney and cardiovascular benefits, and fewer complications from polypharmacy or frailty [19]. SGLT2is are known to cause side effects such as dehydration, UTIs, or genital mycotic infections, which may be more pronounced in older adults, leading to discontinuation [31]. Furthermore, older individuals frequently take multiple medications, increasing the complexity of managing an additional drug such as an SGLT2is. In one study included in the analysis, we observed that patients using GLP-1RAs and SGLT2is were significantly younger (57.0 vs. 61.7 years, *p* < 0.001) which underscores key trends in treatment patterns [19]. Improving the prescribing rates among older patients requires multifaceted approaches addressing clinical concerns, patient education, and system-level barriers. Clinician education and decision support are essential to reframe the perception that older adults universally experience more side effects. Providers should be informed that while risks such as volume depletion exist, they are not inevitable and can be mitigated with proper patient selection and monitoring. Additionally, emphasizing the cardiovascular and renal benefits of SGLT-2is beyond glycemic control can help shift prescribing habits. Implementing clinical decision support tools within electronic health records (EHRs) can prompt appropriate prescribing and ensure eligible patients receive these therapies. Patient-centered education and shared decision-making play a crucial role in dispelling misconceptions about SGLT-2is, such as exaggerated concerns over UTIs and falls. Individualized discussions should focus on weighing benefits against potential risks, starting with lower doses, encouraging adequate hydration, and monitoring kidney function and electrolytes to enhance safety. Providing accessible educational materials can empower patients to recognize the benefits and manage mild side effects proactively. At the system level, financial and logistical barriers should be addressed. Aligning quality metrics and incentives with guideline-directed medical therapy (GDMT) can encourage prescribing, particularly within Medicare plans. Simplifying insurance coverage and formulary access through patient assistance programs and Medicare Part D improvements can also enhance affordability. Encouraging interdisciplinary collaboration—involving pharmacists, primary care physicians, and specialists—can facilitate optimal medication selection and monitoring.

In addition to age, the disparity of initiation largely rests upon providers to take a more holistic approach when treating the patients. Recent data show a potential solution could be targeting specific providers in the treatment paradigm that are more equipped to address patient’s needs [32]. Primary care providers face a wide range of issues during each visit, and endocrinologists see only one in six patients with type 2 diabetes (T2D) and atherosclerotic cardiovascular disease [32]. In contrast, 70% of patients with T2D and high cardiovascular risk are managed in cardiology outpatient clinics, making cardiologists particularly well-positioned to drive the adoption of SGLT2is and GLP-1RAs [32]. Nevertheless, cardiologists’ adoption of these treatments has been slow [32]. A 2019 survey from a large U.S. health system revealed that 80.6% of cardiologists had not prescribed an SGLT2i in the past year, and 83.9% had not prescribed a GLP-1RA [32]. Additionally, nearly two-thirds of the cardiologists surveyed cited a lack of knowledge about how to prescribe these medications safely, and more than half felt that prescribing them was not within their responsibility [32]. One solution recommended is provider education when it comes to the need for the implementation of these agents and the disparities that exist around minority populations. Furthermore, education is crucial for boosting adoption, but a substantial body of evidence indicates that it alone is insufficient to drive lasting changes in clinician behavior. Most studies show that a combination of education, along with audit and feedback, is what ultimately leads to significant improvements in healthcare quality [33].

Race can play a critical role in the initiation of SGLT2is and GLP-1RAs, influenced by disparities in healthcare access, prescribing practices, and socioeconomic factors. Individuals from racial and ethnic minority groups, such as Black, Hispanic, Asian, and Native American populations, may face reduced access to healthcare resources, including access to specialists (e.g., endocrinologists or nephrologists) who are more likely to prescribe SGLT2is. Geographic and systemic inequities, including fewer pharmacies or healthcare facilities in underserved areas, may limit access to these medications as well. In addition, studies included in this analysis have shown that clinicians may be less likely to prescribe advanced diabetes therapies to racial minorities, possibly due to unconscious biases or assumptions about adherence or cost concerns. Lastly, racial and ethnic minorities have been under-represented in clinical trials of these medicines [27]. As a result, there may be less confidence among providers in prescribing these medications to certain populations due to gaps in evidence on safety and efficacy in diverse groups. In this analysis, one study found pharmacy dispensing of SGLT2is was lower among American Indians and Alaska Natives, Black, and Hispanic patients with type 2 diabetes, and pharmacy dispensing of GLP-1RAs medications was lower among all patients with type 2 diabetes from minority groups in six US large healthcare delivery systems [20]. Improving the uptake of these medications among Black, Hispanic, and other minority patients requires addressing systemic barriers to access and expanding pharmacy-based interventions. Affordability remains a major challenge, as many Black and Hispanic patients face cost concerns, lack of insurance coverage, and formulary restrictions that limit access to these medications. Expanding Medicaid coverage, patient assistance programs, and advocating for generic alternatives can help reduce financial barriers. Additionally, healthcare systems should work to streamline prior authorization processes, particularly within Medicare and Medicaid, to prevent unnecessary delays in prescribing. Community-based health initiatives and partnerships with federally qualified health centers (FQHCs) can also play a critical role in increasing access in underserved areas. Pharmacists serve as trusted healthcare providers in many Black and Hispanic communities, making them essential for increasing SGLT-2i or GLP1-RA uptake. Expanding pharmacist-led interventions in community pharmacies can improve awareness and access by providing medication counseling, addressing adherence concerns, and assisting with affordability programs. Beyond pharmacies, faith-based organizations, local barbershops, and community health fairs can serve as additional platforms for engaging patients and providing culturally relevant education on the benefits of these medications. By integrating pharmacists and community leaders into prescribing and education efforts, more patients can gain access to these life-saving medications.

When looking at sex in this analysis, men were prescribed SGLT2is and GLP-1RAs at higher rates for several reasons. The primary reason was the side effect profile of these medicines being better tolerated by men, especially with SGLT2is [34]. Women are more prone to certain side effects of SGLT2is, such as genital mycotic infections (e.g., yeast infections), which are more common in women due to anatomical and hormonal differences [34]. The presence of a history of urinary tract infections may deter providers from initiating therapy in women, particularly those with a history of recurrent infections [34]. Providers may weigh these risks more heavily when considering SGLT2is for women, leading to lower prescription rates. When it comes to GLP-1RAs, common side effects are nausea, vomiting, and gastrointestinal discomfort. Providers may be more cautious about prescribing these medications to women, who might report or tolerate such side effects differently than men [35]. In this analysis, it was found that the female sex is independently associated with lower rates of SGLT2i use, which highlights significant sex disparities in prescribing patterns and treatment uptake [22]. The independent association between the female sex and lower rates of SGLT2i use underscores a critical sex disparity in diabetes and cardiovascular care. This discrepancy arises from a combination of clinical concerns about side effects, sex bias in risk assessment, and systemic barriers to access.

For risk factors such as education level, providers typically have a perceived ability for patients to be able to understand and manage treatment [36,37]. These medications often require patient education regarding their benefits and potential side-effects. Providers may perceive patients with higher education levels as better equipped to understand and manage these complexities, leading to higher prescription rates. In addition, patients with higher education levels are often more engaged in discussion about their care, asking questions, and understanding advanced treatment options which may make providers more confident to prescribe more expensive medications. Health literacy plays a crucial role in this dynamic, as patients with higher health literacy are better able to comprehend medical information and actively participate in decision-making about their treatment. These understanding fosters trust and collaboration between patients and providers, potentially influencing prescribing practices. More educated patients are traditionally associated with a healthier lifestyle, which, in addition to treatment, improves treatment success. Lastly, we found that patients living in neighborhoods with higher education levels were more likely to receive newer kidney and cardiovascular protective medications [8].

Despite the strong evidence supporting these medicines for managing comorbidities like HF, CKD, and ASCVD, their usage remains lower than expected. Several factors contribute to this gap, including prescribing habits, patient misconceptions, and fragmented care [38]. Providers may continue to prescribe older or more familiar treatments (e.g., ACE inhibitors, ARBs, or beta-blockers for HF) instead of newer therapies like SGLT2is and GLP-1RAs. Patients may perceive SGLT2is and GLP-1RAs as being primarily for diabetes/weight loss and may not recognize their broader benefits for HF, CKD, and ASCVD, leading to lower usage and adherence. Lastly, coordination between multiple providers may be suboptimal, leading to missed opportunities to prescribe or reinforce the use of SGLT2 inhibitors and GLP-1RAs [39].

When it comes to insurance and income, it is evident that both are important when trying to afford high-cost medications. In our analysis, patients with comprehensive private insurance were more likely to afford SGLT2i and GLP-1RAs due to better drug coverage and lower out-of-pocket costs [1]. Patients without insurance are significantly less likely to use SGLT2is or GLP-1RAs due to the prohibitive cost, often resorting to cheaper, older medications like metformin, sulfonylureas, or insulin [40]. In this analysis, individuals with higher incomes are more likely to afford medications, either through better insurance plans or the ability to pay out-of-pocket.

The quality of the studies included significantly impacts the interpretation of our findings. The predominance of low-quality evidence, as assessed by the STROBE criteria, weakens the robustness of our conclusions. The STROBE checklist serves as a critical tool for evaluating study design, analysis, and reporting, ensuring methodological rigor. Its inclusion in this scoping review allows for a systematic assessment of study quality, despite this review’s broad scope. Many studies failed to meet essential STROBE standards, reinforcing the need for caution in interpreting results and highlighting the need for well-designed, high-quality research adhering to established reporting guidelines.

### Strengths and Limitations

This scoping review demonstrates several notable strengths. It comprehensively explores an important public health concern by examining disparities in the uptake of SGLT2is and GLP-1RAs, particularly among high-risk populations such as Black and minority groups. By mapping evidence related to disparities, risk factors, and gaps, this review offers valuable insights into health equity challenges in diabetes care and cardio-renal protection. A rigorous and systematic methodology further strengthens this review’s reliability. Searches were conducted across major databases, including PubMed, Google Scholar, Scopus, MEDLINE, and the AI-powered search engine Consensus, ensuring broad literature coverage. The use of dual independent screening and data extraction processes minimized selection bias and improved the accuracy of study inclusion. Another strength of this review is its focus on multiple risk factors. By analyzing disparities across various dimensions such as race, age, sex, education, comorbidities, insurance, and income, this review provides a holistic understanding of the multifaceted barriers influencing medication uptake. This approach highlights structural, provider-related, and patient-level contributors to underutilization. Additionally, this review effectively identifies key research gaps, including the absence of proposed solutions for increasing adoption of SGLT2is and GLP-1RAs. This review also offers critical insights into provider behavior, including differences between specialist and primary care prescribing patterns and the impact of provider education. These findings inform strategies to improve clinician adoption of these medications. Finally, this review is particularly timely, focusing on studies published between 2019 and 2024. This timeframe reflects the recent emergence of data on disparities, underscoring the growing attention to health equity issues in diabetes and cardiovascular care.

This review has several limitations that must be acknowledged. One of the primary concerns is the limited study quality. Among the 19 studies included, none were graded as high-quality, and more than half were rated as low-quality based on the STROBE criteria, which weakens the overall strength of the conclusions (Table 3). Additionally, the predominance of retrospective cohort designs (63.2%) poses a challenge in establishing causal relationships and determining associations between risk factors and medication uptake (Table 1). Another significant limitation is the narrow scope of solutions. While this review successfully identifies disparities in the uptake of SGLT2is and GLP-1RAs, none of the included studies proposed actionable solutions to address these gaps, highlighting a critical shortfall in the current literature regarding intervention strategies. The geographic restriction of the review further limits its applicability. By focusing exclusively on studies conducted in the US, the findings may not be generalizable to other healthcare systems where socioeconomic conditions, healthcare access, and disparities vary in comparison with the US. Additionally, this review reflects an under-representation of key factors that could provide further insights into medication uptake. For instance, the impact of education level was only examined in 4 out of 19 studies, leaving a gap in understanding its broader influence on prescribing patterns and adherence. Furthermore, patient preferences, particularly regarding injectable therapies like GLP-1RAs, were not explored in any of the studies. This omission limits the understanding of how patient hesitancy or preferences may act as barriers to the uptake and adherence of these medications.

This scoping review offers valuable insights into the disparities in SGLT2i and GLP-1RA uptake among minority populations, underscoring the role of race, age, sex, education, and socioeconomic factors. While the strengths include a rigorous methodology and comprehensive analysis of risk factors, the findings are limited by the low quality of included studies, limited geographic scope, and lack of proposed solutions. Future research should address these gaps by exploring actionable interventions, conducting higher-quality studies, and expanding the analysis to include diverse racial and ethnic populations, patient preferences, and global perspectives.

## 6. Conclusions

From this comprehensive analysis, it is evident that the adoption of SGLT2is and GLP-1RAs, despite their proven benefits for kidney and cardiovascular protection, remains suboptimal due to a variety of factors. Age, sex, education, race, insurance, and income all influence prescribing practices and patient uptake, creating disparities in treatment access and utilization. Addressing these gaps requires a multi-faceted approach, including improved provider education, patient engagement, and systemic healthcare reforms to enhance access and affordability. By leveraging targeted education, robust care coordination, and efforts to reduce socioeconomic and racial inequities, the adoption of these advanced therapies can be improved, ensuring that all eligible patients can benefit from their life-saving potential.

## Figures and Tables

**Table 1:** Inclusion and exclusion criteria.

Inclusion Criteria	Exclusion Criteria
Articles pertained to the disparities of initiation and/or risk factors associated with the initiation of SGLT2is and/or GLP-1RAs	Articles only examined obesity and not CKD, HF, or DM
Articles included were required to be available in English	Articles were not published in English
Population of interest in the study was in the United States	Articles were published as opinion papers, commentaries, conference abstracts, reviews, or meta-analyses
	Articles related to pediatric population (age < 16 years)

**Table 2:** Characteristics of included studies [8,9,10,11,12,13,14,15,16,17,18,19,20,21,22,23,24,25,26].

Characteristics of Included Studies		
N = 19	Number	Percentage
**Year of Publication (n)**		
2024	5	26.3
2023	3	15.7
2022	5	26.3
2021	5	26.3
2019	1	5.3
**Study design**		
Retrospective Cohort Study	12	63.2
Cross-Sectional Study	7	36.8
**Journal Published**		
*Journal of Managed Care & Specialty Pharmacy*	2	10.6
*Diabetes Care*	4	21
*Cureus*	1	5.3
*Journal of General Internal Medicine*	1	5.3
*The Journal of the American Medical Association*	4	21
*The American Journal of Cardiology*	1	5.3
*The Lancet Regional Health*	1	5.3
The Public Library of Science	1	5.3
*Journal of Diabetes and Its Complications*	1	5.3
The American College of Cardiology	1	5.3
*Diabetes Technology & Therapeutics*	1	5.3
*Kidney Medicine*	1	5.3

**Table 3:** Summary of key findings from included studies [8,9,10,11,12,13,14,15,16,17,18,19,20,21,22,23,24,25,26].

	Corresponding Author	Study Design	Patient Population	Key Findings	Key Risk Factors	General Limitations
1	Deborah O Ogunsanmi [9]	Retrospective cohort study	7723	Patients with cerebrovascular comorbidities were less likely, while those with obesity or from higher-education neighborhoods were more likely, to be prescribed SGLT2is/GLP-1RAs.	Age, Race, Sex, Education, Comorbidties, Income	The study used data from one large healthcare system, limiting its generalizability to other populations or regions.
2	Julie M. Paik [14]	Retrospective cohort study	160,489	Among SGLT2i initiators, more had baseline HF or ischemic heart disease. SGLT2i use in DKD patients decreased with worsening kidney function, while GLP-1RA use increased.	Age, Provider Type, Comorbidies, Income	The study included only commercially insured and Medicare Advantage patients, limiting generalizability to uninsured patients and other healthcare systems.
3	Daniel Antwi-Amoabeng [12]	Cross-Sectional study	2746	Ethnicity, income, and insurance type impact SGLT2i and GLP-1RA prescriptions. Patients on these medications have better mortality outcomes than those on other treatments.	Age, Race, Insurance, Income	The study used data from one large healthcare system, limiting its generalizability to other populations or regions.
4	Lurit Bepo [11]	Cross-Sectional study	4997	Racial and ethnic disparities in SGLT2i/GLP-1RA use vary by insurance type, with the largest gaps in private insurance persisting after adjustments for cardiovascular, socioeconomic, and access factors. Disparities were lowest among Medicaid enrollees.	Race, Education, Comorbidies, Insurance, Income	Sample size limitations in the MEPS cohort prevented further disaggregation of race/ethnicity in adjusted analyses.
5	Rozalina G. McCoy [24]	Retrospective cohort study	382,574	Racial/ethnic disparities in GLP-1RA use were observed, with Black, Hispanic, and Asian patients less likely to initiate treatment than White patients. While Black and Hispanic patients showed no disparity in SGLT2i initiation after adjusting for demographics and clinical factors, Asian patients remained less likely to start SGLT2is.	Age, Provider Type, Race, Sex, Comorbidies, Insurance, Income	The study used data from a single national health insurer, limiting generalizability to other private and public insurance plans.
6	Faraz S. Ahmad [8]	Retrospective cohort study	22,672	Non-Hispanic Black patients had lower odds of receiving SGLT2is or GLP-1RAs. Overall, prescription rates were low, with racial disparities evident.	Provider Type, Race, Sex, Comorbidies, Insurance	The study identified racial and ethnic disparities in prescribing but could not fully examine social determinants like systemic barriers and structural racism.
7	Lauren A. Eberly [13]	Retrospective cohort study	1,180,260	GLP-1RA use was low, even among patients with ASCVD. Asian, Black, and Hispanic patients, as well as those from lower-income areas, had lower GLP-1RA use, reflecting inequities in prescribing.	Provider Type, Race, Sex, Income	The study could not fully evaluate the impact of structural racism, historical mistreatment, and discrimination on healthcare access and delivery.
8	HoJin Shin [23]	Retrospective cohort study	549,755	SGLT2i and GLP-1RA use as first-line treatment remained low but steadily increased, especially in patients with existing CVD.	Age, Provider Type, Comorbidies	The findings reflect data from 2013 to 2019, a period of evolving clinical guidelines and prescribing practices that may have influenced the observed trends.
9	L.A. Rodriguez [20]	Retrospective cohort study	687,165	Pharmacy dispensing of SGLT2is was lower among Black and Hispanic patients with type 2 diabetes, while GLP-1RA dispensing was lower among all minority groups across six large U.S. healthcare systems.	Age, Race, Sex, Comorbidies, Insurance, Income	The study’s findings may not generalize to uninsured or underinsured populations or regions beyond the six integrated healthcare systems, limiting its relevance to national trends and health equity.
10	Jingchuan Guo [16]	Retrospective cohort study	795,469	Non-Hispanic Black, American Indian, Hispanic, and Asian patients were less likely than non-Hispanic White patients to initiate SGLT2is/GLP-1RAs. Dual Medicare–Medicaid enrollees also had lower initiation rates.	Age, Race, Comorbidies, Insurance	The study focused on Medicare fee-for-service beneficiaries aged 65+, limiting its applicability to younger populations or those with private insurance.
11	Lauren A. Eberly [22]	Retrospective cohort study	934,737	Black race, female sex, and lower household income were independently associated with lower SGLT2i use, with inequities also seen among patients with HFrEF, ASCVD, and CKD.	Provider Type, Race, Sex, Comorbidies, Insurance, Income	The dataset mainly included patients from the South and Midwest, limiting its generalizability to other regions and uninsured populations.
12	Julio A. Lamprea-Montealegre [15]	Cross-sectional study	1,197,880	Patients with severe albuminuria were less likely to receive SGLT2is or GLP-1RAs than nonalbuminuric CKD patients. Higher ASCVD (≥20% vs. <5%) and ESKD (≥5% vs. <1%) risk were both associated with lower odds of SGLT2i prescription.	Provider Type, Comorbidies	The study uses Veterans Health Administration (VHA) data, where uniform pharmacy benefits apply, limiting generalizability to healthcare systems with significant co-payments and medication cost barriers.
13	Benjamin G. Mittman [6]	Retrospective cohort study	4777	Patients on GLP-1RAs/SGLT2is were younger, more likely to be White, privately insured, college-educated, higher-income, and had higher BMI but lower rates of hypertension and CKD than those on other drug classes.	Age, Race, Sex, Education, Comorbidies, Insurance, Income	Potential misclassification of diabetes type may have occurred, as some individuals with type 1 diabetes might have been included due to missing diagnostic criteria in the dataset.
14	Leila R. Zelnick [10]	Cross-Sectional study	1375	Disparities in medication use were seen by age, race, and insurance status. Older adults used fewer SGLT2is and GLP-1RAs. Non-Hispanic Black and Mexican American participants with CKD, CHF, or ASCVD had lower SGLT2i use, while GLP-1RA use was lower overall in these groups. Uninsured individuals had lower GLP-1RA use.	Age, Provider Type, Race, Sex, Education, Comorbidies, Insurance, Income	Income estimates for the 2017–2020 cycle relied on 2017–2018 data, potentially limiting the accuracy of income distribution over the full study period.
15	Julio A. Lamprea-Montealegre [21]	Cross-Sectional study	1,197,914	Prescription rates of SGLT2is and GLP-1RAs were low across all racial and ethnic groups compared to White patients. Hispanic patients had significantly lower odds of receiving these medications, even after adjusting for individual and system-level factors.	Provider Type, Race, Comorbidies, Insurance	The study relies on Veterans Health Administration (VHA) data with uniform pharmacy benefits, limiting generalizability to other healthcare systems with variable medication cost-sharing structures.
16	Salim S.Virani [19]	Cross-Sectional study	105,799	SGLT2i use was lower among older adults, female patients, and Black patients.	Age, Provider Type, Race, Sex, Comorbidies, Insurance	The study population was limited to Veterans Health Administration (VHA) patients, restricting generalizability to non-VHA populations.
17	Sara J. Cromer [26]	Cross-Sectional study	4,057,725	Older age was linked to lower, and male sex to higher, likelihood of GLP-1RA or SGLT2i initiation. Non-Hispanic Black and other racial/ethnic groups, along with greater socioeconomic deprivation, also had lower initiation rates.	Age, Provider Type, Race, Sex, Comorbidies	The study focused on Medicare fee-for-service beneficiaries, excluding those with Medicare Advantage or other insurance, limiting generalizability to the broader U.S. population.
18	Rozalina G. McCoy [25]	Retrospective cohort study	1,054,727	Younger, healthier, non-Black patients with commercial insurance were most likely to start SGLT2is, while those with MI, HF, kidney disease, or prior hypoglycemia were less likely.	Age, Provider Type, Race, Comorbidies, Insurance	The study used data from a single large U.S. health plan covering private and Medicare Advantage patients, limiting generalizability to uninsured individuals or those in other healthcare systems.
19	Julie Z. Zhao [17]	Retrospective cohort study	53,029	Racial/ethnic disparities were observed in SGLT2i and GLP-1RA initiation, with Black patients significantly less likely to receive these medications.	Age, Race, Sex, Comorbidies, Income	The study identified disparities in treatment initiation but did not assess patient- or provider-level barriers, such as cost or provider bias.

Abbreviations: SGLT2is: sodium/glucose cotransporter-2 inhibitors; GLP-1RAs: glucagon-like peptide-1 receptor agonists; HF: heart failure; DM: diabetes mellitus; DKD: diabetic kidney disease.

**Table 4:** Quality assessment [8,9,10,11,12,13,14,15,16,17,18,19,20,21,22,23,24,25,26].

Reference	Authors	Assessment	Reference	Authors	Assessment
[9]	Ogunsanmi et al., 2003	●●	[16]	Guo et al., 2024	●
[14]	Paik et al., 2021	●	[13]	Eberly et al., 2021	●●
[12]	Antwi-Amoabeng et al., 2024	●	[15]	Lamprea-Montealegre et al., 2022	●
[11]	Bepo et al., 2024	●	[6]	Mittman et al., 2024	●
[24]	McCoy et al., 2021	●●	[10]	Zelnick et al., 2022	●●
[8]	Ahmad et al., 2023	●	[21]	Lamprea-Montealegre et al., 2022	●●
[22]	Eberly et al., 2021	●●	[19]	Virani et al.,	●
[23]	Shin et al., 2021	●	[26]	Cromer et al., 2023	●●
[20]	Rodriguez et al., 2024	●●	[25]	McCoy et al., 2019	●
[18]	Zhao et al., 2023	●●			

STROBE Quality: High (>25) = ●●●, Medium (20–25) = ●●, Low (<20) = ●.

## Data Availability

The original contributions presented in this study are included in the article. Further inquiries can be directed to the corresponding authors.
